# Integrated analysis of gene expression from carbon metabolism, proteome and metabolome, reveals altered primary metabolism in *Eucalyptus grandis* bark, in response to seasonal variation

**DOI:** 10.1186/s12870-016-0839-8

**Published:** 2016-07-01

**Authors:** Ilara Gabriela Frasson Budzinski, David H. Moon, Júlia Silva Morosini, Pernilla Lindén, Juliano Bragatto, Thomaz Moritz, Carlos Alberto Labate

**Affiliations:** Laboratório Max Feffer de Genética de Plantas, Departamento de Genética, Escola Superior de Agricultura Luiz de Queiroz, Universidade de São Paulo, Piracicaba, SP 13418-900 Brasil; Umeå Plant Science Centre, Department of Forest Genetics and Plant Physiology, Swedish University of Agricultural Sciences, Umeå, SE-901 83 Sweden

**Keywords:** *Eucalyptus grandis*, Metabolomics, Primary metabolism, Proteomics, RT-qPCR

## Abstract

**Background:**

Seasonal variation is presumed to play an important role in the regulation of tree growth, especially for *Eucalyptus grandis*, a fast-growing tree. This variation may induce changes in the whole tree at transcriptional, protein and metabolite levels. Bark represents an important group of tissues that protect trees from desiccation and pathogen attack, and it has been identified as potential feedstock for lignocellulosic derived biofuels. Despite the growing interest, little is known about the molecular mechanisms that regulates bark metabolism, particularly in tropical countries.

**Results:**

In this study we report the changes observed in the primary metabolism of *E. grandis* bark during two contrasting seasons in Brazil, summer (wet) and winter (dry), through the combination of transcripts (RT-qPCR), proteome (2-DE gels) and metabolome (GC-MS) analysis, in an integrated manner. Twenty-four genes, involved in carbon metabolism, were analyzed in the two seasons. Eleven were up-regulated in summer, three were up-regulated in winter and ten did not show statistical differences in the expression pattern. The proteomic analysis using 2-DE gels showed 77 proteins expressing differences in abundance, with 38 spots up-regulated in summer and 37 in winter. Different metabolites significantly accumulated during winter.

**Conclusions:**

This study revealed a metabolic reconfiguration in the primary metabolism of *E. grandis* bark, triggered by seasonal variation. Transcripts and protein data suggests that during winter carbohydrate formation seems to be favored by tree metabolism. Glucose, fructose and sucrose accumulated at significant levels during the winter.

**Electronic supplementary material:**

The online version of this article (doi:10.1186/s12870-016-0839-8) contains supplementary material, which is available to authorized users.

## Background

Eucalyptus species are the most widely planted hardwood due to the quality of its wood. These fast-growing trees are cultivated under a range of different climates and can be destined to different industrial processes (e.g. pulp and paper, charcoal, fuel wood, and solid wood products). Most of the current Eucalyptus production in Brazil is cultivated in an area over 5.4 million hectares [1]. Given its fast growing rates and coppicing ability, eucalyptus has also been identified as a potential feedstock for biofuels [2]. Besides, bark is also a source of nutrients, carbon as well as being used to form a protective covering of soils in commercial plantations. Bark comprises all the tissues outside the vascular cambium and it includes primary and secondary phloem, cortex, first periderm, rhytidome and tissues formed by dilatation growth [3]. These tissues also protect woody plant organs and healing tissues from dehydration, solar irradiation and pathogens [4]. Bark formation is initiated by the process of cell division at the cambium, which produces xylem on the inner woody side and phloem, the primary bark tissue, on the exterior bark side. The phloem tissue contains phloem parenchyma, phloem fibers, companion cells and sieve cells [5]. Although tree development is regulated by seasonal periods, little is known about the underlying molecular processes related to growth, especially in the bark. Soler et al. [4] analyzed the seasonal variation in mRNA abundance in cork tissue from *Quercus suber*. They found transcripts for structural genes involved in suberin production accumulating in late spring; this accumulation was significantly correlated with temperature and relative humidity. The increased expression of genes involved in stress was also strongly correlated to temperature. Using the proteomic approach, Pagter et al. [6] observed distinct seasonal protein patterns in bark of *Hydrangea macrophylla* and *Hydrangea paniculata* during cold acclimation and de-acclimation.

In the present study, we investigated the metabolic response of *E. grandis* bark in two contrasting seasons: summer/wet and winter/dry, using RT-qPCR, proteomic and metabolomic analyses, with emphasis on carbon metabolism. Despite the importance of bark metabolism to the whole tree and its potential for biofuel production [7], to our knowledge this is the first molecular study showing changes in eucalyptus bark metabolism, in response to seasonal variation.

## Results and discussion

### Seasonality influences mRNA expression in bark

**Fig. 1:**
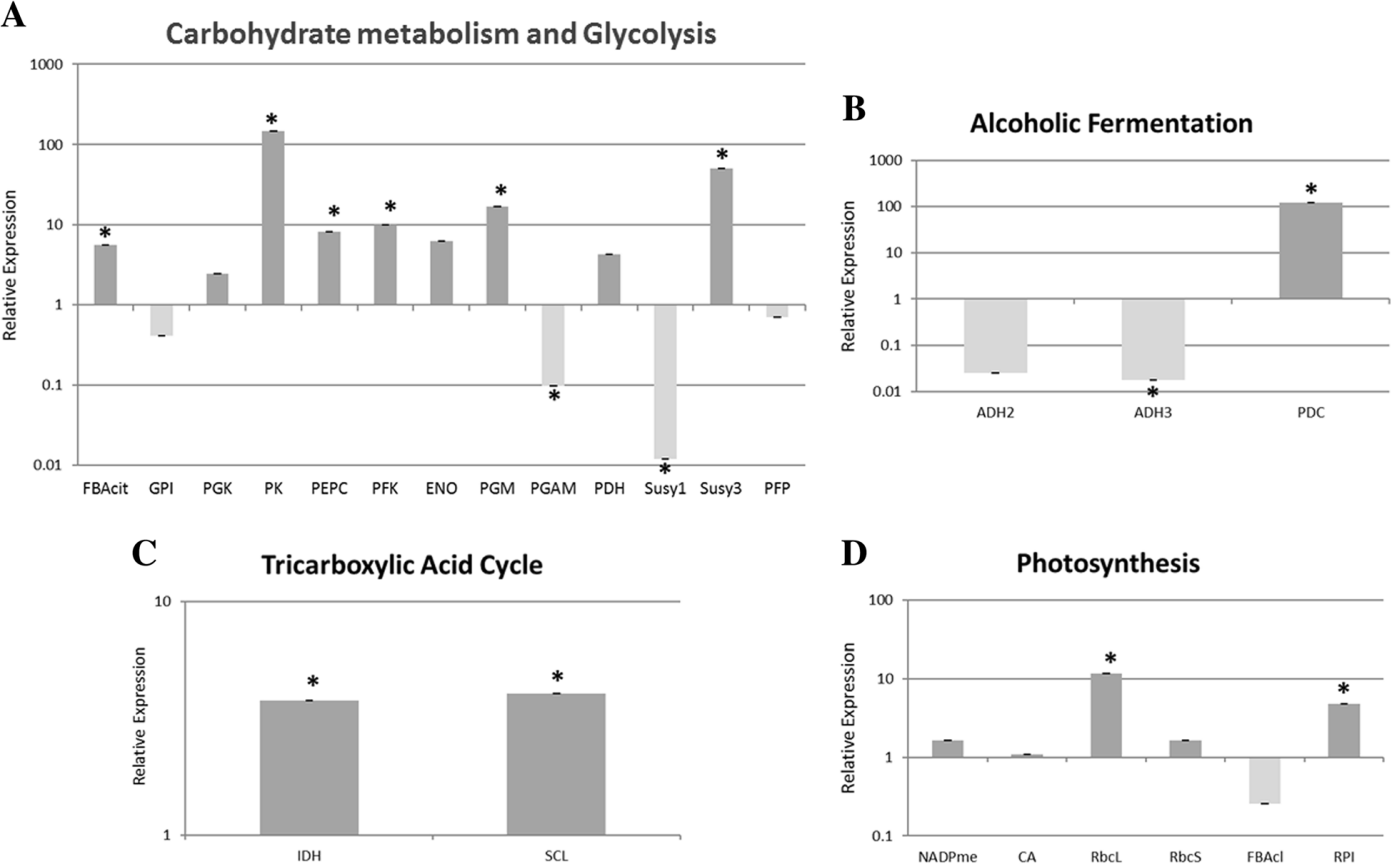
Seasonal variation of transcripts involved in primary metabolism (**a**–**d**), in *E. grandis* bark, by RT-qPCR. Data are expressed as log fold change and winter values were used as a control. Expression was determined relative to α-tubulin and MDHc (Material and Methods). Asterisks indicates genes that are significantly expressed (*P* ≤ 0,05). Abbreviations: FBAcyt (fructose bisphosphate aldolase cytoplasmatic); GPI (glucose-6-phosphate isomerase); PGK (phosphoglycerate kinase); PK (pyruvate kinase); PEPC (phosphoenolpyruvate carboxylase); PFK (ATP-dependent phosphofructokinase); ENO (enolase); PGM (phosphoglucomutase); PGAM (phosphoglyceratemutase); PDH (pyruvate dehydrogenase); SuSy1 (sucrose synthase 1); SuSy3 (sucrose synthase 3); PFP (PPi-dependent phosphofructokinase); ADH2 (alcohol dehydrogenase 2); ADH3 (alcohol dehydrogenase 3); PDC (pyruvate decarboxylase); IDH (isocitrate dehyidrogenase); SCL (succinyl-coa ligase); NADP-ME (NADP malic enzyme); CA (carbonic anhydrase); RbcL (rubisco large subunit); RbcS (rubisco small subunit); FBAcl (fructose bisphosphate aldolase chloroplastidial) and RPI (ribose-5-phosphateisomerase). Three biological replicates, each with three technical replicates were analyzed per sample and error bars are standard errors of mean

As a first step to understand the molecular mechanisms underlying the maintenance of primary metabolism in *E. grandis* bark during the summer and winter, the relative mRNA abundance of a set of 24 candidate genes (Additional file 1) involved in carbohydrate metabolism with emphasis in glycolysis, sucrose metabolism, ethanol fermentation, tricarboxylic acid cycle and carbon fixation were analyzed by RT-qPCR. Figure 1 shows the relative mRNA abundance of these genes during the contrasting seasons. Of these, 11 genes were up-regulated in summer (Fructose bisphosphate aldolase cytoplasmatic (*FBAcyt*), Pyruvate kinase (*PK*), Phosphoenolpyruvate carboxylase (*PEPC*), ATP-dependent phosphofructokinase (*PFK*), Phosphoglucomutase (*PGM*), Sucrose synthase 3 (*SuSy3*), Pyruvate decarboxylase (*PDC*), Isocitrate dehydrogenase (*IDH*), Succinyl-CoA ligase (*SCL*), Rubisco large subunit (*RbcL*) and Ribose 5-phosphate isomerase (*RPI*)), three genes were up-regulated in winter (Phosphoglycerate mutase (*PGAM*), Sucrose synthase 1 (*SuSy*1) and Alcohol dehydrogenase 3 (*ADH*3)) and 10 genes showed no statistically significant differences between seasons (Glucose 6-Phosphate isomerase (*GPI*), Phosphoglycerate kinase (*PGK*), Enolase (*ENO*), Pyruvate dehydrogenase (*PDH*), PPi-dependent phosphofructokinase (*PFP*), Alcohol dehydrogenase 2 (*ADH2*), NADP Malic enzyme (*NADP-ME*), Carbonic anhydrase (*CA*), Rubisco small subunit (*RbcS*) and Fructose bisphosphate aldolase chloroplast (*FBAcl*)).

Most of the genes involved in carbohydrate metabolism and glycolysis were up-regulated in summer (Fig. 1a), suggesting that during this season, primary metabolism is being required for the production of reducing power and ATP, necessary for the development and growth of the trees. During winter, the metabolic activity decreases and, as a consequence, trees reduce their growth rates, thus reducing their energy consumption. It is interesting to note that each SuSy analyzed showed a different expression pattern; *SuSy*1 was highly expressed in winter while *SuSy*3 were highly expressed in summer. Susy plays a crucial role in the sucrose metabolism. The enzyme catalyzes the reversible conversion of sucrose and UDP to UDP-glucose and fructose, but it also degrades sucrose [8]. Thus, each alternative transcript could be required for a different molecular function. Moreover, it has been proposed that there are two forms of Susy in higher plants [9], one is a soluble enzyme found in the cytoplasm (S-Susy) and the other is a membrane associate enzyme (P-Susy). Both forms are probably regulated by the phosphorylation status of the enzyme [10]. Further investigations are necessary to completely understand the mechanism that regulates Susy isoforms in eucalyptus bark.

The genes *ADH*3 and *PDC* involved in the fermentative metabolism showed statistically significant differences in expression (Fig. 1b). Curiously, each transcript was up-regulated in a different season. As we expected *ADH3* was up-regulated in summer, during the growing season. We also expected that *PDC* was up-regulated in summer; however, it showed an opposite expression pattern. As a fast growing tree, eucalyptus requires high levels of ATP in summer to maintain this process. At the same time, the cambial and bark tissues are under hypoxic conditions due to anatomical barriers to gas exchange. Thus, respiration might shift from the aerobic to the ethanolic fermentation mode, as a means to maintain substrate-level for ATP production. This requires transcriptional activation of the essential genes of ethanolic fermentation, *PDC* and *ADH* [11]. Our data suggest that ethanolic fermentation is probably required in summer and winter, with the participation of different *PDC* alternative transcripts. The regulatory role of *PDC* in ethanolic fermentation has not yet been fully explained [12]. In Arabidopsis there are four genes encoding PDC and a microarray dataset related to low oxygen conditions, revealed that *PDC1* and *PDC2* were strongly up-regulated under low oxygen, whereas *PDC3* and *PDC4* mRNA levels were not induced by anoxia, suggesting that these two genes were unlikely to play a role during anoxic stress [13].

Yang et al. [14] found one *ADH* in the bark of *Robinia pseudoacacia,* however the authors did not discuss the transcript function. The study of the ethanolic fermentation process in trees started around the late 1980s [15–17] although until now little information is available and more research in this field is necessary.

*IDH* and *SCL* were the genes from TCA analyzed in our work, and both were up-regulated in summer (Fig. 1c). *IDH* is responsible to the oxidative decarboxylation of isocitrate to 2-oxoglutarate, the function of this enzyme has been associated with the maintenance of the 2-oxoglutarate level and the regulation on nitrogen assimilation [18]. *SCL* catalyzes the reversible interconversion of succinyl-CoA to succinate, characterization of the regulatory properties of this enzyme suggests that it may represent an adaptive mechanism in the attempt to maintain the rate of respiration under suboptimal condition [19, 20]. Our results may suggest that in summer, the pyruvate produced *via* glycolysis is mainly sent to mitochondria, where it will be used by the TCA cycle, for energy production, instead of being used in the fermentative metabolism. It’s important to mention that as described above, most of the transcripts analyzed in carbohydrate metabolism and glycolysis were also up-regulated in summer, indicating that this pathway is working in the direction of pyruvate formation. Trees show higher metabolic activity in summer and during this period, active growth, glycolysis and the TCA cycle are fundamental to maintain high metabolic rate. Curiously, we observed that *PEPC* was up-regulated in summer. The export of TCA cycle intermediates requires the importation of substrates that can generate both acetyl-CoA and oxaloacetate (OAA) [21]. If pyruvate is used as a unique substrate, export of TCA cycle intermediates, would reduce OAA regeneration and bring the TCA cycle to a halt. In such condition PEPC plays an important role in the anaplerotic fixation of CO_2_ and in the route to replenish TCA intermediates that are withdrawn from the pool [22, 23]. PEPC activity was reported as being more than ten times higher in stem of *Fagus sylvatica* than in leaves [23]. This high PEPC activity in stem could be explained by the anaplerotic roles of PEPC, common in C3 plants or, as in C4 metabolism, PEPC could be supplying malate for NADP-ME, which supplies CO_2_ for Rubisco by malate descarboxylating [23].

In trees several physiological process such as growth, respiration, the lack of stomata and the low permeability of stem peridermal layers to gaseous diffusion, result in a high internal CO_2_ concentration ([CO_2_]) (range <1 to 26 %) and thus 500–800 times higher CO_2_ levels than in ordinary plant organs or ambient air [23, 24]. High CO_2_ concentrations effectively eliminate photorespiration, and enhance photosynthetic potential within branches and bark [25]. In accordance with this, we found in eucalyptus bark the accumulation of mRNAs related to CO_2_ fixation (Fig. 1). Among the analyzed transcripts only *RbcL* and *RPI* were differentially expressed, they were up-regulated in summer. The *NADP-ME* transcript was not differentially expressed between summer and winter. *RbcL* was up-regulated in summer, the season in which trees have the highest respiration levels, leading to an increase in CO_2_ release in the interior of the stem. The *RPI* was also up-regulated in summer, it catalyzes the conversion of ribose 5-phosphate into ribulose 5-phosphate in the Calvin cycle and pentose phosphate pathways [26]. Considering the high CO_2_ availability inside the bark, our results suggests that *RbcL* and *RPI* are acting in the refixation of metabolically produced CO_2_. The stems of woody plants possess greenish tissues, that contain chlorophylls (the chlorenchymes) and are localized below the outer peridermal or rhytidomal layers. These tissues are able to use the stem internal CO_2_ and the light penetrating the rhytidome to fixate carbon [27], explaining the presence of rubisco in the bark. Thus, the high CO_2_ concentration and the low oxygen availability can explain the fact that *RbcL* was differentially expressed and *RbcS* was not. Under such rich CO_2_ environment, inside the bark, maybe it is unnecessary for the plant to over produce *RbcS* to promote CO_2_ fixation. Higher plant Rubisco is composed of eight large subunits coded for by a single gene, the *RbcL*, and eight small subunits coded for by the nuclear *RbcS* multigene family [28]. The RbcL contain the catalytic site of the enzyme and is responsible for the carboxylase and oxygenase reactions but the *RbcS*, whose precise role in structure and function of Rubisco remains poorly understood, contributes to the differences in kinetic properties among Rubisco enzyme [29]. Transcripts of enzymes that participates in the photosystems I and II were also found in *Robinia pseudoacacia* bark [14].

### Proteomic Analysis of *E. grandis* bark during summer and winter

The analysis of the protein profile from *E. grandis* bark was initially evaluated by 2-DE gels, using strips with pH 3–10 in the first dimension (data not shown). The majority of the proteins spots observed were concentrated in the pH range 4–7 (data not shown). Based on this, we decided to analyze the protein profile, in triplicates, using the range of pH 4–7 (Additional file 2: Figure S1). After image analysis, 445 and 424 protein spots were identified in summer and winter gels, respectively. From these, 125 spots were differentially expressed (*P* ≤ 0.05) between seasons and all of them were analyzed by mass spectrometry (Additional file 2: Figure S1). Among them 75 proteins spots (63 %) were successfully identified in the databank (Table 1); 38 were up-regulated in summer and 37 in winter. The remaining proteins were not further considered in the analysis as they did not match the search criteria.

**Table 1:** Identification of differentially expressed proteins spots from 2-DE gels

Spot n°	Protein	Protein score	Coverage %	Sequence	N° of Peptides	Fold change (summer/winter)
	1.1.1.2 C1 Metabolism					
21	RuBisCO large subunit-binding	410	11 %	Egrandis_v1_0.005399 m	6	0.4
58	Ribulose bisphosphate carboxylase large chain	325	15 %	RBL_ANTFO	6	0.54
59	RuBisCO large subunit-binding	263	13 %	Egrandis_v1_0.005399 m	4	0.74
73	Formate dehydrogenase	1297	39 %	Egrandis_v1_0.015998 m	11	1.93
7	Phosphoglycerate kinase	3928	58 %	Egrandis_v1_0.014782 m	16	0.48
	1.1.2 Energy Metabolism (Carbon)					
8	Enolase	122	5 %	Egrandis_v1_0.021648 m	1	0.46
14	Phosphoglycerate kinase	1681	46 %	Egrandis_v1_0.014782 m	12	0.3
51	Phosphoglycerate kinase	1452	32 %	Egrandis_v1_0.014782 m	8	0.4
52	Enolase	128	3 %	Egrandis_v1_0.010202 m	5	3.77
60	Triosephosphate isomerase	1092	45 %	Egrandis_v1_0.020110 m	9	0.56
101	Enolase	1610	19 %	ENO1_HEVBR	6	1.33
114	Enolase	929	15 %	ENO1_HEVBR	4	0.43
	1.1.3 Energy Transfer/Atp-Proton Motive Force					
3	HSP20-like chaperones superfamily protein	153	28 %	Egrandis_v1_0.029820 m	2	3.98
25	ATPase, V1 complex, subunit B	964	27 %	Egrandis_v1_0.010528 m	10	0.35
30	ATP synthase alpha/beta family protein	1664	51 %	Egrandis_v1_0.007569 m	17	0.36
46	Citrate synthase	286	21 %	Egrandis_v1_0.011280 m	7	1.85
91	Citrate synthase	523	18 %	Egrandis_v1_0.011280 m	5	1.56
97	Malate dehydrogenase	1094	30 %	Egrandis_v1_0.018951 m	6	0.62
	1.2.1 Amino AcidMetabolism					
71	Phosphoglycerate dehydrogenase	456	35 %	Egrandis_v1_0.014972 m	7	0.54
	1.2.3 Nucleotide/Nucleoside and Nucleotide Sugar Metabolism					
2	UDP-sugar pyrophosphorylase	395	10 %	Egrandis_v1_0.005961 m	3	0.48
41	Adenosinekinase	1965	45 %	Egrandis_v1_0.018389 m	10	1.96
61	UDP-glucose pyrophosphorylase	89	6 %	Egrandis_v1_0.011888 m	2	2.08
63	UDP-glucose dehydrogenase	554	22 %	Egrandis_v1_0.010940 m	6	1.9
103	UDP-glucose pyrophosphorylase	621	32 %	Egrandis_v1_0.011888 m	7	0.49
106	UDP-glucose pyrophosphorylase	1187	41 %	Egrandis_v1_0.011888 m	13	0.46
	1.2.8 Secondary Metabolism					
87	NAD(P)-linked oxidoreductase	445	24 %	Egrandis_v1_0.019083 m	5	2.05
96	Phenylcoumaranbenzylicether reductase	2400	76 %	Egrandis_v1_0.020543 m	15	0.45
	2.1 Cell Processes					
78	Vacuolar H + -ATPasecatalyticsubunit	933	20 %	Egrandis_v1_0.006156 m	8	0.41
70	14-3-3 protein	654	26 %	Egrandis_v1_0.043757 m	6	0.45
123	14-3-3- protein	1031	39 %	Egrandis_v1_0.023614 m	7	0.49
	2.2.2 Protection Responses/Detoxification					
1	Ascorbate peroxidase	1788	41 %	Egrandis_v1_0.024254 m	6	4.71
24	Peroxidase	209	9 %	gi|242089639	2	1.9
31	Ascorbate peroxidase	84	7 %	Egrandis_v1_0.024254 m	1	0.64
33	Ascorbate peroxidase	1406	41 %	Egrandis_v1_0.024164 m	6	0.49
45	Ascorbateperoxidase	121	10 %	Egrandis_v1_0.024164 m	1	1.93
47	Glutathioneperoxidase	75	8 %	Egrandis_v1_0.024172 m	2	4.22
69	Glutathione S-transferase, C-terminal-like	157	8 %	Egrandis_v1_0.022631 m	2	0.52
74	Ascorbateperoxidase	1624	57 %	Egrandis_v1_0.024217 m	6	4.11
79	Ascorbateperoxidase	1447	51 %	Egrandis_v1_0.024254 m	7	3.28
99	Ascorbateperoxidase	200	14 %	Egrandis_v1_0.024254 m	2	2.32
107	Copper/zinc-superoxidedismutase	296	19 %	Egrandis_v1_0.029096 m	2	1.42
	2.2.3.2 Abiotic					
90	Late embryogenesisabundantprotein	1384	43 %	Egrandis_v1_0.020038 m	13	0.55
	4.1.2.4 LigninMetabolism					
105	Caffeicacid 3-O-methyltransferase	383	31 %	COMT1_EUCGU	7	0.49
124	Caffeicacid 3-O-methyltransferase	328	3 %	Egrandis_v1_0.003388 m	6	0.57
	4.1.2.5 Expansins, Xetand Extensin					
54	Major Latex protein MLP-like	1055	50 %	Egrandis_v1_0.029781 m	6	0.71
95	Major Latex protein (MLP-like)	792	55 %	Egrandis_v1_0.029781 m	6	0.83
	4.3 Cytoskeleton					
17	Beta-tubulin	267	13 %	Egrandis_v1_0.012369 m	4	0.42
102	Beta-tubulin	1533	27 %	Egrandis_v1_0.012451 m	11	0.46
111	Beta-tubulin	2034	34 %	Egrandis_v1_0.012369 m	15	0.79
	5.2 Rna Related					
44	Glycine-rich RNA-binding protein	283	21 %	Egrandis_v1_0.020038 m	4	0.36
	5.3.3 Translation Related					
66	Eukaryoticinitiationfactor	381	9 %	Egrandis_v1_0.014208 m	3	2.56
75	Elongationfactor 1 beta	684	14 %	Egrandis_v1_0.021389 m	3	0.34
84	Eukaryoticinitiationfactor	1471	41 %	Egrandis_v1_0.014208 m	13	1.83
	5.3.5 Protein Folding/Chaperoning					
4	Heatshockproteins	565	28 %	Egrandis_v1_0.021389 m	5	1.58
11	Heatshockprotein	2052	28 %	Egrandis_v1_0.044829 m	15	2.53
13	Heatshockprotein	230	13 %	Egrandis_v1_0.027871 m	2	0.32
40	Heat shock cognate 70 kDa protein	1888	31 %	Egrandis_v1_0.003388 m	18	2.72
57	Heatshockproteinmitochondrial	385	21 %	Egrandis_v1_0.026204 m	3	1.89
62	Heatshockprotein (HSP20)	1331	50 %	Egrandis_v1_0.045806 m	9	2.99
67	Heatshockprotein	157	15 %	Egrandis_v1_0.029182 m	2	4.11
76	Heatshockprotein (Hsp20)	1589	26 %	Egrandis_v1_0.021389 m	7	1.52
82	Heatshock 70 kDaprotein	3155	44 %	Egrandis_v1_0.005502 m	23	3.84
94	Heatshockprotein	810	34 %	Egrandis_v1_0.029182 m	5	1.54
98	Heatshockprotein (HSP20)	363	23 %	Egrandis_v1_0.026663 m	4	1.8
104	Heatshockprotein	383	18 %	Egrandis_v1_0.026663 m	5	1.99
112	Heatshockprotein	2682	47 %	Egrandis_v1_0.025526 m	9	0.53
118	Heatshockcognate 70 kDa	1292	24 %	Egrandis_v1_0.003884 m	14	0.53
120	Heatshockprotein	1010	22 %	Egrandis_v1_0.044829 m	11	1.84
125	Heat shock 70 kDa protein, mitochondrial	1413	29 %	Egrandis_v1_0.004853 m	13	1.66
	5.3.6 Protein Cleavage and Turnover					
42	Proteasomalregulatoryprotein	187	14 %	Egrandis_v1_0.018412 m	4	1.97
55	Proteasomesubunit beta type	1215	34 %	Egrandis_v1_0.021324 m	7	1.78
68	26S proteasome non-ATPase regulatory particle	533	28 %	Egrandis_v1_0.023175 m	4	0.63
	6.2 Putative Protein					
116	NAD(P)-binding Rossmann-fold superfamily protein	386	21 %	Egrandis_v1_0.018330 m	6	0.72

### Functional classification of proteins differentially expressed found in bark

**Fig. 2:**
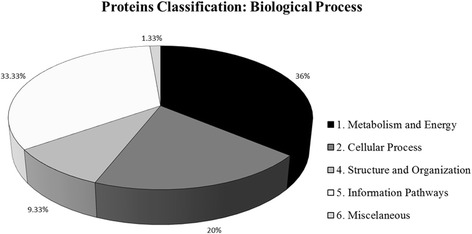
Categorization of differentially expressed proteins in *E. grandis* bark in two contrasting seasons

The differentially expressed proteins between summer and winter were classified according to their biological processes into 5 categories (Fig. 2), similarly to the convention used by Rison et al. [30] and Carvalho et al. [31]. Proteins representing the functional categories “1-Metabolism and Energy” (36 %), “5-Information Pathway” (33.3 %) and “2-Cellular Process” (20 %) were the most abundant. Proteins from “1-Metabolism and Energy” category were distributed into five subcategories (Carbon Metabolism, Energy Metabolism, Energy Transfer/ATP-proton motive force, Nucleotide/Nucleoside Metabolism and Secondary Metabolism). The same was observed for the proteins from the categories “5-Information Pathways” (subcategories Translation Related, Protein Folding/Chaperoning and Protein Turnover”) and “2-Cellular Process” (Signal Transduction and Response Proteins and detoxification).

### Proteins identified related to the primary metabolism

Surprisingly we found only few proteins differentially expressed acting in primary metabolism. This result can be due to limitations of 2-DE gels technique or by the fact that only a reduced number of proteins related to primary metabolism changed in abundance between the two seasons. We found different proteins acting in carbohydrate metabolism, cell wall biosynthesis, glycolysis and TCA: UDP-glucose pyrophosphorylase (UGPase), UDP-glucose dehydrogenase (UGDH), phosphoglycerate kinase (three probable isoforms), triosephosphate isomerase, enolase (four probable isoforms), citrate synthase (two probable isoform) and malate dehydrogenase, all of them belong to different subcategories from the category “1. Metabolism and Energy”.

Four probable isoforms of UGPase were found, showing different expression patterns. Three of them were up-regulated in winter and the last one was up-regulated in summer, indicating temporal expression regulation. UGPase is a key enzyme in sucrose metabolism that catalyzes the reversible production of glucose-1-phosphate and UTP to UDP-glucose and pyrophosphate, depending on the metabolic status of the tissue. In photosynthetic tissues UGPase converts glucose-1-phosphate to UDP-glucose, which can be utilized for sucrose synthesis, or cell wall polysaccharides [32]. In non-photosynthetic sink tissues, UGPase is linked to sucrose degradation pathways by converting UDP-glucose produced by sucrose synthase to glucose-1-phosphate [33]. UGDH was up-regulated in winter and converts UDP-glucose to UDP-glucuronate, which is a precursor of hemicellulose and pectin. In woody tissues, the role of UGPase and UGP is poorly understood. Two UGDH genes mainly expressed in roots, stem and bark of 6-month-old *E. grandis* were cloned [34].

We found tree probable isoforms of phosphoglycerate kinase and four possible isoforms of triosephosphate isomerase, all of them up-regulated in winter. Two enolases were also up-regulated in winter and two were up-regulated in summer. Changes in abundance of proteins related to carbohydrate and energy metabolism during cold acclimation were observed in the *Hydrangea paniculata* bark [6]. In the subcategory “Carbon Metabolism” we found tree RbcL isoforms, all of them up-regulated during winter. The RbcS was not found in our work, it is possible that RbcS was not detected as a differentially expressed spot. To our knowledge there are no data in literature related to the rubisco subunits expression pattern (transcripts and proteins) in trees bark. In the *E. grandis* cambial zone of trees with different ages, it has been demonstrated the presence of RbcL, by immunoblotting, the RbcS subunit was only detected in the leaf control [35]. Proteins related to carbon fixation were found in the bark of *Prunus persica* [36] and *Picea sitchensis* [37]. The protein profile of RbcL observed in our work was the opposite of the transcriptional pattern that we found, as the transcript was up-regulated in summer. The discrepancy between transcriptomic and proteomic data is widely discussed in the literature and probably indicates the occurrence of post-transcriptional and/or post-translational modifications [38–40].

### Proteins identified related to other biological pathways

Proteomics provides the identification of a set of proteins expressed at a specific time, tissue or condition. Thus, we identified a diverse range of proteins involved in other biological process, besides primary metabolism. The proteins implicated in Protein folding/Chaperoning were the most abundant, among them 17 heat shock (HSP) were found and two were up-regulated in winter. Some HSPs are molecular chaperones that regulate the folding, localization, accumulation, and degradation of protein [41]. Thus HSPs play a crucial role in protecting plants against multiple environmental stresses by re-establishing normal protein conformation and homeostasis. Other proteins related to stress were found, such as: one late embryogenic protein (LEA), seven ascorbate peroxidases (APX) and two 14-3-3 proteins. LEA protein was up-regulated in winter; this protein is found in plant seeds and also in vegetative tissues under stress conditions such as cold, drought, or high salinity [42]. Among the seven APX identified, five were up-regulated in summer and two in winter. APX expression is induced in response to different forms of stress that results in the accumulation of reactive oxygen species (ROS) [43]. 14-3-3 proteins were also found. These are phosphoserine-binding proteins that regulate the activities of a wide array of targets via direct protein–protein interactions. In plants 14-3-3 regulates the plasma membrane H^+^-ATPase and enzymes of carbon and nitrogen metabolism [44]. Three translation-related proteins were identified, two initiation factors (up regulated in summer) and one elongation factor (up regulated in winter). Two caffeic acid o-methyltransferase (COMT) were up regulated in winter. COMT is one of the most important enzymes controlling lignin monomers production in plant cell wall synthesis.

### Soluble sugars in Bark

**Fig. 3:**
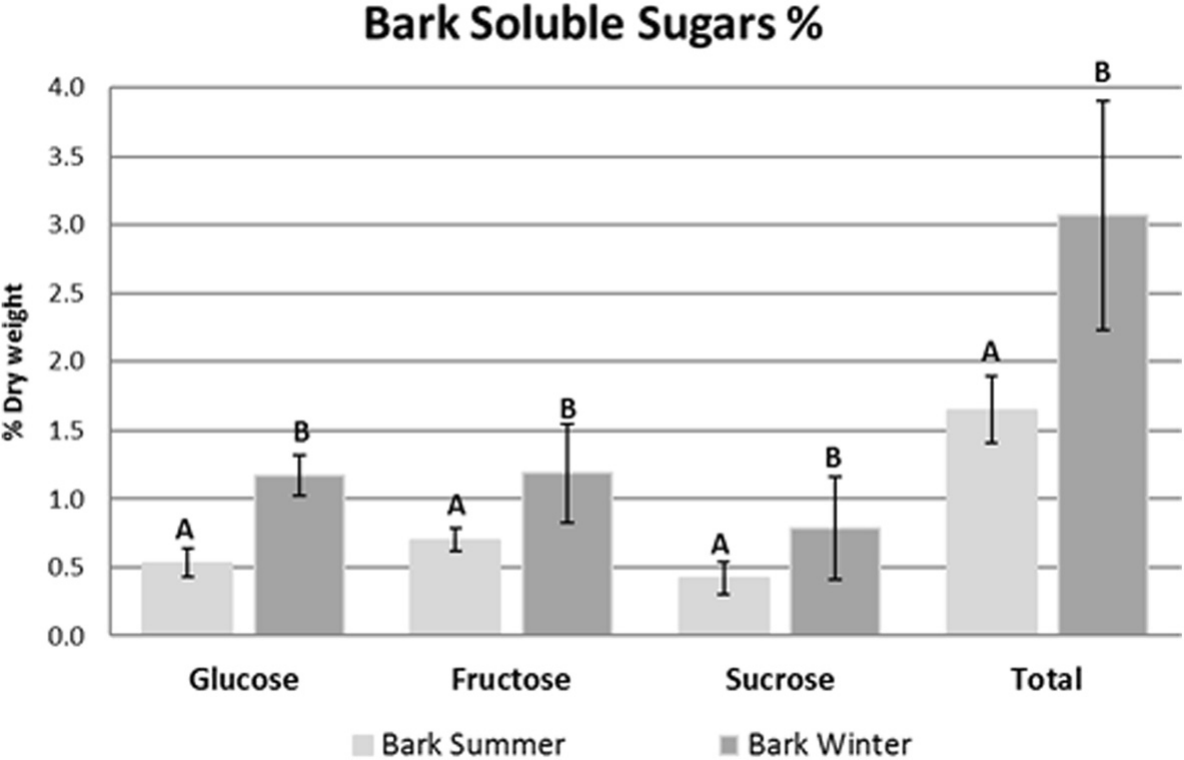
Soluble sugar content of *E. grandis* bark during summer and winter. Soluble sugars were quantified by HPLC (Material and Methods). Three biological replicates with three technical replicates were performed for each season. Bars with same letter are not significantly different, based on Tukey’s test (*P* ≤ 0.05). Error bars are standard errors of mean

Trees store a large amount of carbohydrates in parenchymatous tissues of their wood and bark. These stored carbohydrates can be required to meet the carbon needs for tree maintenance and growth when the current level of photosynthesis is not enough [45]. To confirm differences in carbohydrate storage between summer and winter we measured the soluble sugars from the summer and winter bark samples. A significant increase (*P* ≤ 0.05) in glucose, fructose and sucrose as well as in total soluble sugar content was observed in winter bark’s (Fig. 3). In accordance with the literature our results indicate sugar accumulation during winter, the season in which trees show a reduction in growth (tropical regions) or even dormancy (temperate regions). During the summer season, the water availability is higher and eucalyptus has a higher growth rate. Thus, the consumption of carbohydrates stored in the bark is necessary, in order to maintain the high metabolic rate. In *Cornus sericea L*. glucose, fructose, sucrose and raffinose were the soluble sugars predominant in both, bark and wood tissues in the winter. In the early spring, the soluble sugar concentration decreased and the concentration of starch increased. Soluble sugars increased in the fall and reached a maximum in mid-winter [46]. Higher concentration of soluble sugars was observed in the bark of *Eucalyptus globulus* submitted to water deficit [47]. Carbon investment in storage of carbohydrates may provide safety margins to allow trees to maintain hydraulic transport and metabolism during episodes of stress such as drought and insect attacks [48]. We believed that in our work glucose, fructose and sucrose might be accumulated during winter in response to diminished water availability instead of low temperatures. Thus, differences in precipitation between summer and winter, could be implicating in metabolic changes related to carbon allocation. Another point to be considered is the soluble sugar accumulation in response to low temperatures, as a mechanism of cold acclimation [6, 49] specially in trees from temperate regions. Travert et al. [50] exposed two genotypes of *Eucalyptus* cell-suspension cultures to low temperatures. The resistant cells (hybrid *Eucalyptus* gunnii x Eucalyptus *globules*) accumulated soluble sugars, in particular sucrose and fructose. In contrast the frost-sensitive cells (hybrid *Eucalyptus cypellocarpa* x *Eucalyptus globules*) did not accumulate soluble sugars in response to the same treatment. The authors correlated these data to the potential involvement of several carbohydrates (glucose, fructose, sucrose, raffinose and manitol) for improving freezing tolerance in Eucalyptus cells as well as the cryoprotection by sugars during cold acclimation.

### Metabolic profiling during seasonal variation

**Fig. 4:**
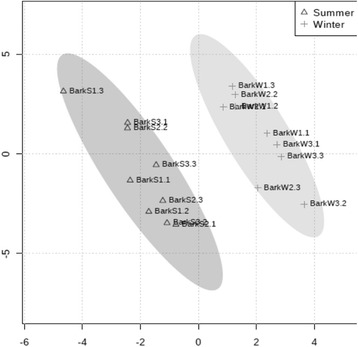
PLS-DA scores plot showing a significant separation (*R* ^2^ = 0.95 and *Q* ^2^ = 0.96) between *E. grandis* summer and winter barks. Component 1 and 2 contributes with 33.3 % and 30.9 %, respectively, of the total variance. Three biological replicates, each with three technical replicates were analyzed per sample

Metabolic profiling was performed by GC-MS to identify changes in the primary metabolites resulting from seasonal variation. In total, 32 metabolites were identified in *E. grandis* bark. All metabolites were classified into five categories and the most representative were organic acids (28 %), sugars (21 %), fatty acids (18 %) and amino acids (9 %) (Additional file 1). A PLS-DA (partial least square discriminant analysis) derived scores plot (Fig. 4) showed a statistically significant separation of these two groups. The two PLS components accounted for 64.2 % of the total variance. Our PLS-DA model comparing summer and winter barks had *R*^2^ and *Q*^2^ values of 0.95 and 0.91, respectively. To identify the variables that had the most significant contribution in discriminating between metabolite profiles of summer and winter samples, we considered the variable importance in the projection (VIP) values higher than 1.0 combined with *p*-value less than 0.05. Based on it, we found eight metabolites differentially abundant between the summer and winter groups (Table 2). The organic acids highly abundant in bark during winter were shikimate and dehydroascorbic acid. Shikimate is an intermediate in the shikimic pathway, which links the carbohydrate metabolism to biosynthesis of aromatic compounds [51]. Dehydroascorbic acid is an oxidized form of the important antioxidant vitamin C [52]. The flavonoid taxifolin and the sugar substitute erythritol were also highly abundant in winter. Malate, galactinol, gluconate and fumarate were the metabolites highly abundant during summer. Malate and fumarate are important intermediates in the TCA. In some C3 plants they can be accumulated during the day, decreasing during the night, suggesting that they function as transient carbon storage molecules [53]. In C3 plants, photosynthetic cells surrounding the vascular system are supplied with malate or CO_2_ from the xylem vessels. Thus, malate could be decarboxylated by these cells bordering the vascular system and the CO_2_ could be used in photosynthesis to produce carbohydrates [54]. Although soluble sugars (sucrose, glucose and fructose) accumulated in winter, galactinol was highly abundant during summer. It participates in the raffinose biosynthesis and both of them act protecting cellular membranes from abiotic stresses [55].

**Table 2:** Metabolites differentially abundant in bark

Metabollite	Class	VIP	*p*.value	↑abundant
Erythritol	Sugar Alcohol	2.85	5.40E-08	winter
Shikimate	Organic acid	1.96	2.50E-05	winter
Malate	Organic acid	1.9	4.90E-05	summer
Galactinol	Sugar	1.52	3.50E-04	summer
Gluconate	Sugar	1.29	1.60E-02	summer
Dehydroascorbic acid	Organic acid	1.25	8.50E-03	winter
Fumarate	Organic acid	1.14	1.70E-02	summer
Taxifolin	Flavonoid	1.01	3.30E-02	winter

### Integrated analyses of *E. grandis* bark in response to seasonal variations

**Fig. 5:**
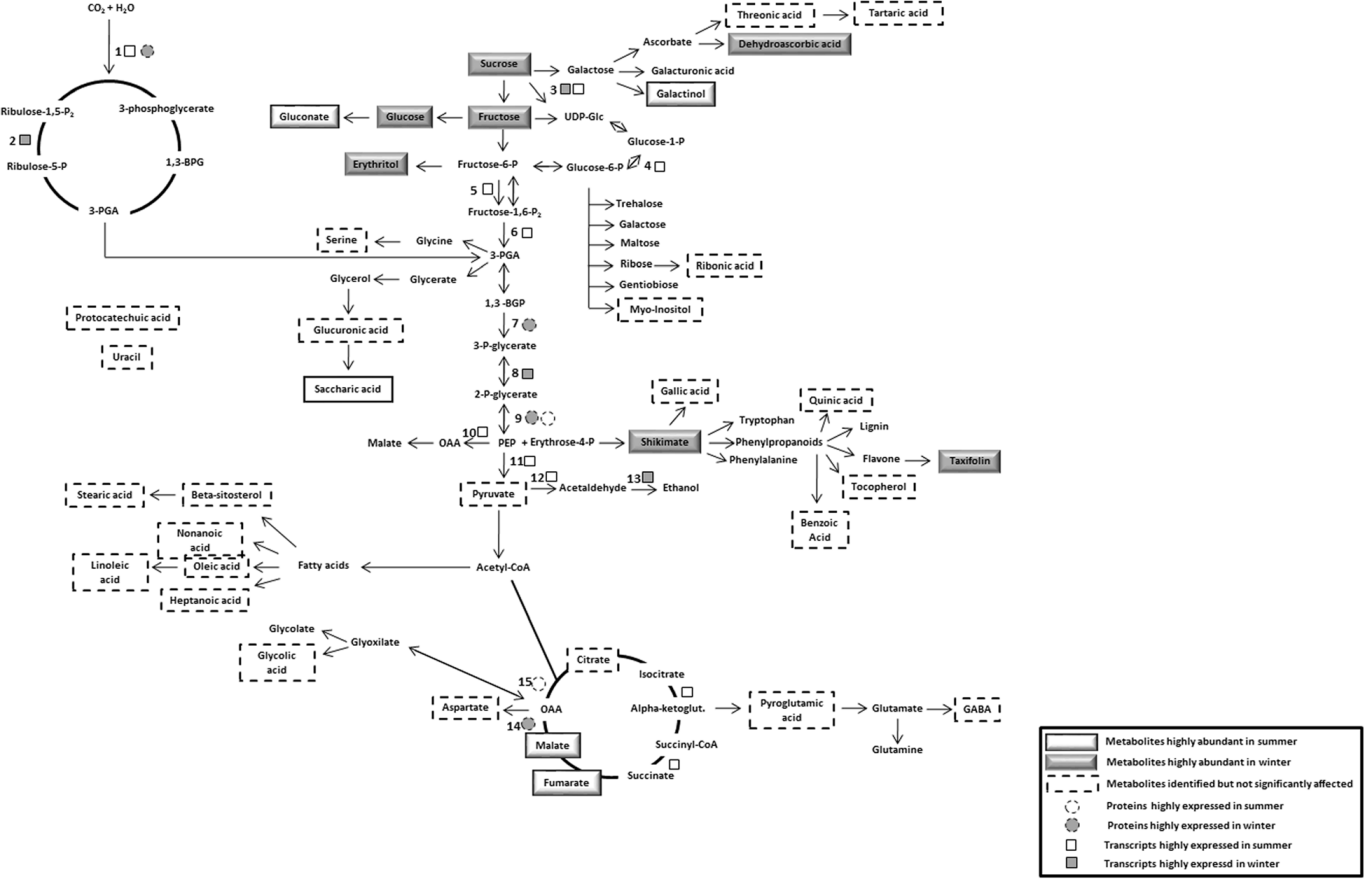
Differentially expressed transcripts, proteins and metabolites involved in the bark primary metabolism. The transcripts (*square*), proteins (*circles*) and metabolites (boxes) shown in white were up-regulated in summer, and those in gray were up-regulated in winter. Metabolites in dashed boxes were detected but were not significantly affected by seasonal changes (summer/winter). 1- Ribose-5-phosphate isomerase (RPI), 2- Rubisco large subunit (RbcL), 3- Sucrose synthase (SuSy), 4- Phosphoglucomutase (PGM), 5- ATP-dependent phosphofructokinase (PFK), 6- Fructose bisphosphate aldolase (FBAcyt), 7- Phosphoglycerate kinase (PGK), 8-Phosphoglycerate mutase (PGAM), 9- Enolase (ENO), 10-Phosphoenolpyruvate carboxylase (PEPC), 11- Pyruvate kinase (PK), 12- Pyruvate decarboxilase (PDC), 13- Alcohol dehydrogenase (ADH), 14- Isocitrate dehydrogenase (IDH) and 15- Succinyl-CoA ligase (SCL)

Bark plays a fundamental role in transporting assimilated carbon to sink tissues where it can be used for growth and/or storage [56]. To obtain a more holistic view about the dynamic changes occurring in *E. grandis* bark metabolism due to seasonal variation, all the information generated in our work was grouped (Fig. 5). Our data demonstrate differences in carbon partitioning between summer and winter samples. At the transcriptional and metabolic level carbohydrate formation seems to be favored in winter and some proteins related to this pathway were also up-regulated in this season. During winter, the dry season, tree growth diminishes and, as a consequence, lower levels of carbon skeletons are required to promote cell wall growth and enlargement, contributing to sugar accumulation. Besides, in opposite to what happens in trees, from temperate regions, we do not believe that carbohydrates accumulate in *E. grandis* bark, in response to cold acclimation/freezing tolerance. We believe that this is due to lower water availability.

We did not find proteins related to ethanolic fermentation, probably due to limitations in 2D-gels, on the other hand we found differential expression for *ADH* and *PDC* transcripts. *ADH*3 was up-regulated in summer and *PDC* was up-regulated in winter. Although we did not find any metabolite directly related to carbon fixation, transcripts and proteins acting in Calvin-Benson Cycle were identified, especially RbcL, strongly suggesting that carbon fixation is occurring in bark.

## Conclusions

Studies about the metabolism of bark trees are scarce and the effect of seasonal variation is also poorly understood. Given the importance of *Eucalyptus*, especially in Brazil, for different industrial applications, it is important to comprehend how seasonal variations affect the whole tree and tissues. Our results strongly suggest a metabolic reconfiguration triggered by the shift between summer and winter periods, as we found significant differences in all levels investigated (transcripts, proteins and metabolites). During summer, when trees are fast growing, energy compounds are necessary to support glycolysis and mitochondrial electron transport chain. However, the high respiration rates associated with anatomical barriers generate a hypoxic environment inside the bark. Thus, ethanolic fermentation is an important pathway regenerating NAD^+^ to the maintenance of glycolysis and plant metabolism. It is known that bark is a storage tissue and that during winter tree growth diminished. In winter, soluble sugars accumulate probably because of the diminished water available in tropical regions, and not because of lower temperatures (cold acclimation) as observed in temperate trees. An interesting data we observed was the identification of RbcL transcripts and proteins in bark. This study provides important data to understand seasonal variation in *Eucalyptus* bark. Future studies are necessary to identify which isoforms are involved in ethanolic fermentation and carbohydrate metabolism and also to clarify the precise function of RbcL in bark.

## Methods

### Plant material and experimental conditions

Tissue samples were harvested from commercial clonal trees of six years-old *Eucalyptus grandis*, kindly provided by Suzano Papel e Celulose. The field-trial was situated in Itapetininga city, State of São Paulo, Brazil (23°35′20″ S, 48°03′11″ W) at an altitude of 656 m. To analyze the changes in transcripts, proteins and metabolites during summer/active growth compared to winter/diminished growth, bark samples were harvested in January/2009 and July/2009 for summer and winter, respectively. Samples were harvested in the morning, between 9 and 10:00 am. The average temperature and precipitation during January/summer were 23.7 °C and 213.2 mm, respectively. During the month of July/winter these parameters were 16.8 °C and 47.7 mm, respectively. The bark of each tree was removed at chest height (1.30 m, exposing an area of approximately 20 × 15 cm^2^). The cambial zone tissues in the inner surface of the bark were scraped with a razor blade and discarded, the bark samples were immediately frozen in liquid nitrogen. The field trial was a completely randomized design. Six bulked samples (10 trees each) were used as biological replicates. Three bulks represented summer and three represented winter trees. The plant material was used with the permission of Suzano Papel e Celulose S/A.

### RNA extraction and mRNA isolation

Total RNA was extracted from bark samples using the protocol described by Zeng and Yang [57]. Total RNA concentration was measured spectrophotometrically at 260/ 280 nm, in a U-3300 spectrophotometer (Hitachi, Tokyo, Japan). The absence of RNA degradation was verified by electrophoresis on a formamide-formaldehyde denaturing agarose gel (1 %). mRNA was isolated using Dynabeads® mRNA purification kit (Invitrogen Dynal, Oslo, Norway), according to the manufacturer’s instructions.

### Real-Time PCR

Gene-specific primers were designed with Primer 3 software (Additional file 3: Table S1). Primer pairs were designed as follows: primer length between 18 pb - 25 bp, product length of 100–250 bp, melting temperatures 55–60 °C, GC% between 40 and 60 %. First and second strand cDNA synthesis were performed using the *SuperScriptTM One-Step RT-PCR Platinum® Taq* (Invitrogen, Carlsbad, CA, USA) kit with RT/Platinum®*Taq* (Invitrogen, Carlsbad, CA, USA) and using primers specific for the genes of interest (Additional file 3: Table S1). The cDNAs were produced in a Gene Amp® PCR System 9700 thermocycler (Applied Biosystems, Foster City, CA, USA) using as annealing temperatures 57 °C. The cDNAs (10^-1^) were used as a template for RT-qPCR assays, carried out in an iQ5 instrument (BioRad) to obtain de threshold quantification cycle (*Cq*) and the amplification efficiencies (*E*). At the end of the PCR cycles, the thermocycler was programmed to perform a denaturation curve. The final volume of each reaction was 20 μL, including cDNA, 10 mM of each primer and 1x Supermix SYBR Green real-time RT-PCR (Invitrogen). A negative control (no cDNA template) was included for every gene. Three biological replicates, each with three technical replicates, were analyzed. The calculation of relative expression ratios was carried out with the Relative Expression Software Tool (REST) [58] using the pairwise fixed reallocation randomization test for the statistical significance (*P* ≤ 0.05) [59]. Reference genes (α-tubulin and citoplasmatic malate dehydrogenase (MDHc)) were identified using *NormFinder* [60]. The software *LinReg* [61] was used to calculate the PCR efficiencies and the C*q* values of each gene analyzed.

### Proteomic analysis

Total protein from bark was extracted by grinding the frozen tissue (4 g) and using the phenolic method according to Hurkman and Tanaka [62], with minor modifications described in Celedon et al. [35]. The tissues were homogenized in 15 mL of extraction buffer (0.7 M sucrose, 0.5 M Tris-HCl, pH 7.5, 50 mM EDTA, 0.1 M KCl, 1 % w/v polyvinylpolypirrolidone (PVPP), 2 % v/v 2-mercaptoethanol, and 2 mM PMSF), by shaking for 30 min at 4 °C. An equal volume of Tris-HCl saturated phenol pH 8.5 was added to the protein suspension. After an additional 30 min of shaking at 4 °C, the phases were separated by centrifugation (10,000 × *g* for 30 min at 4 °C). The phenol phase was recovered and re-extracted with an equal volume of extraction buffer. Proteins were precipitated from the phenol phase by adding 5 vol. of 0.1 M ammonium acetate in methanol and incubated overnight at -20 °C. The samples were then centrifuged (10,000 × *g*, 30 min at 4 °C) and the resulting pellets were washed three times with 0.1 M ammonium acetate in methanol, followed by a wash with acetone. The protein pellet was dried under vacuum at 4 °C and suspended in 1 mL of solubilization buffer (7 M urea, 2 M thiourea, 0.4 % v/v Triton X-100, 50 mM dithiothreitol (DTT)). Proteins were quantified using the Bradford method [63]. Protein samples (700 μg) were mixed with buffer (340 μL) containing 10 mM DTT, 4 % (w/v) CHAPS, 1 % IPG buffer (GE Healthcare, Chalfont St. Giles, UK). Bromophenol blue (1 % w/v) was used to rehydrate for 12 h (20 °C and 50 V) the strips of Immobiline IPG (18 cm -pH 4–7, linear gradient, GE Healthcare, Chalfont St. Giles, UK). Rehydrated strips were isoelectrofocused in an Ettan™ IPGphor II™ (GE Healthcare) for 1 h, starting at 100 V and then 500 V for 1 h, 1000 V for 1 h, 5000 V for 1 h and 8000 until reaching a total of 80,000 V-h. Before the second dimension, strips were kept at room temperature for 15 min in equilibration buffer (6 M urea, 2 % w/v SDS, 50 Mm Tris-HCl, pH 6.8, 30 % v/v glycerol) firstly with 1 % w/v DTT and then with 2.5 % w/v iodoacetamide (IAA) and 0.001 % bromophenol blue. The second dimension was performed in 12 % (w/v) polyacrylamide gels, using a Protean II XI 2-D cell electrophoresis system (GE Helathcare), at 30 mA per gel until the dye reached the bottom of the gel. Three biological replicates were performed for each treatment. Proteins were detected using Coomassie Brilliant Blue G-250 [64]. Gels were incubated for 1 h in a solution containing 40 % (v/v) ethanol and 10 % (v/v) acetic acid, in water. For protein detection the gels were left overnight in staining solution (20 % (v/v) methanol, 10 % (w/v) ammonium sulfate, 10 % v/v phosphoric acid, and 0.1 % (w/v) Coomassie G-250). Gels were imaged using an Image scanner III and Labscan v 7.0 software (GE- Healthcare). Image analysis was performed automatically using the Image Master 2D Platinum software v 7.0 (GE Amersham Bioscience). Spots were detected using a smoothness of 8, minimum area of 15 and a saliency of 40, and spots across gels were matched using 5 landmarks per gel. Matching was performed automatically, and systematically confirmed after one-by-one visual checking: artefacts, or spots that could not be confidently validated as true matches, were disregarded and misalignments were corrected manually when appropriate. Spots were considered reproducible when well resolved in at least two of the three biological replicates. The normalized volumes (% vol.) of the corresponding spots from summer and winter samples were compared to estimate differential expression of proteins during different seasons. To ensure the reproducibility between technical replicates, spots with coefficient of variation higher than 30 % were excluded. To identify spots significantly expressed the data collected from protein spot volumes were subjected to Student’s t-test (*P* ≤ 0,05) in Image Master v 7.0 software. In-gel digestion of proteins was performed as described in Celedon et al. [35] After, peptide mixtures were sequenced by online chromatography using a nano-Acquity UPLC (Waters®) sistem coupled to a Q-TOF Ultima API mass spectrometer (Waters, UK). Mass spectrometer parameters were adjusted according Celedon et al. [35]. Ten microliters of sample were loaded onto a pre-column Symetry MCA C18 5 mm, 5630 mm (Waters) for sample pre-concentration and desalination, followed by peptide separation on an LC column Symmetry C185 mm, 32 × 150 mm (Waters). Peptides were eluted using a linear gradient (10–45 %) of solvent B (95 % (v/v) acetonitrile, 0.1 % (v/v) formic acid in water). The flow rate started with 5 mL/min for the first 15 min, then changing to 2 mL/min for the next 25 min, and back to 5 mL/min in the last 5 min. Solvent A consisted of 5 % v/v acetonitrile, and 0.1 % v/v formic acid in water. All analyses were performed using a positive ion mode at 3 kV needle voltage. The mass range was set from 300 to 2000 *m/z*, and the MS/MS spectra were acquired for the most intense peaks having at least 15 counts.

### Protein identification/MSMS-data analysis

The LC-MS/MS were processed using ProteinLynx v 2.0 (Waters) and Mascot Daemon (Matrix Science, Boston, MA) software, and the sequences searched against an in-house *Eucalyptus* database from Phytozome v1.1 (www.phytozome.net/eucalyptus.php) and NCBI. Combined MS-MS/MS search criteria used were as follows: trypsin digestion; fixed modification set as carbamidomethylation of cystein; variable modification set as methionine oxidation); mass accuracy of 50 ppm for the parent ion and MS/MS mass tolerance of 0.1 Da. According to MASCOT probability analysis, only significant hits (*P* ≤ 0.05) were accepted. A match was considered significant if the peptide had a score higher than 70, based on Perkins et al. [65].

### Metabolic profiling

Bark samples were ground into powder in liquid N_2_ and freeze-dried. Metabolites were extracted according to the method described by Hoffman et al. [66], with minor changes. Approximately 5 mg of dried tissue was mixed with 1 mL of a chloroform-methanol-water mix (6:2:2) containing stable isotope reference compounds [15 ng mL^-1^ each of (^13^C_3_)-myristic acid, (^13^C_4_)-hexadecanoic acid, (^2^H_4_)-succinic acid, (^13^C_5_, ^15^N)-glutamic acid, (^2^H_7_)-cholesterol, (^13^C_5_)-proline, (^13^C_4_)-disodiumketoglutarate, (^13^C_12_)-sucrose, (^2^H_4_)-putrescine, (^2^H_6_)-salicylic acid and (^13^C_6_)-glucose). The metabolite extraction proceeded using a vibration mill set to a frequency of 30 Hz s^-1^ for 3 min, with 3 mm tungsten carbide beads added to each extraction tube to increase the extraction efficiency. The extracts were then centrifuged for 10 min at 14,000 rpm in an Eppendorf centrifuge (model 54178).

After, 100 mL of each supernatant was transferred to a GC-vial and evaporated to dryness. The samples were then derivatized with 30 μL of methoxyamine hydrochloride (15 mg mL^-1^) in pyridine for 16 h at room temperature. Trimethylsilylation was performed by adding 30 μL of N-methyl-N-(trimethylsilyl) trifluoroacetamide (MSTFA) with 1 % TMCS to the samples and incubating them for 1 h at room temperature. After silylation, 30 μL of heptane was added. Samples were analyzed, according to Gullberg et al. [67], using gas chromatography with time-of-flight mass spectrometry (GC/TOF-MS) together with blank control samples and a series of *n*-alkanes (C12–C40), which allowed retention index to be calculated [68]. One microliter of each derivatized sample was injected in splitless mode by a CTC Combi Pal Xt Duo (CTC Analytics AG, Switzerland) auto-sampler in an Agilent 7890A gas chromatograph equipped with a 30 m × 0.25 mm i.d. fused-silica capillary column with a chemically bonded 0.25-μm DB 5-MS UI stationary phase (J&W Scientific, Folsom, CA). The injector temperature was 260 °C, the septum purge flow rate was 20 mL min^-1^ and the purge was turned on after 75 s. The gas flow rate through the column was 1 mL min^-1^, the column temperature was held at 70 °C for 2 min, then increased by 15 °C min^-1^ to 320 °C, and held there for 4 min. The column effluent was introduced into the ion source of a Pegasus HT time-of-flight mass spectrometer (Leco Corporation, St. Joseph, MI, USA). The transfer line and the ion source temperatures were 250 and 200 °C, respectively. Ions were generated by a 70 eV electron beam at an ionization current of 2.0 mA, and 20-30 spectra s^-1^ (30 spectras^-1^ run 1, 20 spectra s^-1^ run 2) were recorded in the mass range 50 − 800 *m*/*z*. The acceleration voltage was turned on after a solvent delay of 290 s. The detector voltage was 1450-1490 V (1450 V run 1, 1490 V run 2). All non-processed MS-files from the metabolic analysis were exported into ChromaTOF 2.12 software (Leco Corporation), in which all manual integrations and metabolite identification were done. All data treatment procedures (base-line correction and chromatogram alignment) were performed using customs scripts [68] in MATLAB. To compare the metabolite changes between summer and winter seasons the normalized data set (to tissue dry weight and internal standards) was Pareto scaled, log transformed and applied to multivariate and univariate analytical methods using the MetaboAnalyst software [69]. The supervised classification method PLS-DA was carried out to discriminate between different groups (summer and winter). PLS-DA model fit was evaluated using the R^2^ and Q^2^ cross-validation performance measures [69], both of which vary between 0 and 1. R^2^, the squared correlation coefficient between the dependent variable and the PLS-DA prediction, provides an indication of the “goodness of fit” (a value between zero and one, where one is a perfect correlation) from the model. Q^2^ provides an indication of “goodness-of-prediction” and is the averaged correlation coefficient between the dependent variable and the PLS-DA predictions. To identify the metabolites that contributed to the separation between the two groups we used the VIP from the PLS-DA model. VIP is a weighted sum of squares of the PLS loadings which indicates the importance of the variable to the whole model. Differential metabolites were selected based on PLS-DA model using a combination of VIP value > 1.0 and *p-*value (*P* ≤ 0.05), by the univariate unpaired, two-tailed Student’s t-test.

### Carbohydrate extraction and HPLC analysis

Soluble sugars (glucose, sucrose and fructose) were extracted from bark samples. Tissues were grinded and freeze-dried for 48 h, after 1 mL of water was added in 0.2 g of dry powder and samples were kept in bath (80 °C) for 1 h. Then, samples were centrifuged for 10 min 16.000 *g*, the supernatant was recovered and stored at -4 °C. Summer and winter samples were analyzed using a high-performance liquid chromatography (ICS 2500, HPLC Dionex) with amperometric detection (ED50) equipped with an autosampler, AS50. Sugars were assigned according to the retention times of standards (sucrose, glucose and fructose). A Carbopac PA-1 column (4 × 250 mm, Dionex) and a guard Carbopac PA-10 column (4 × 50 mm, Dionex) were used. To identify statistical differences between summer and winter samples a Tukey’s test (*P* ≤ 0.05) was performed in SAS (SAS Institute, Cary, NC, USA).

## Abbreviations

2-DE, two-dimensional chain electrophoresis; ADH2, alcohol dehydrogenase 2; ADH3, alcohol dehydrogenase 3; ATP, Adenosine triphosphate; CA, carbonic anhydrase; C*q*, threshold quantification cycle; ENO, enolase; FBAcl, fructose bisphosphate aldolase chloroplast; FBAcyt, fructose bisphosphate aldolase cytoplasmatic; GC-MS, gas chromatography mass spectrometry; GPI, glucose 6-phosphate isomerase; IDH, isocitrate dehydrogenase; NADP-ME, NADP Malic enzyme; PDC, pyruvate decarboxylase; PDH, pyruvate dehydrogenase; PEPC, phosphoenolpyruvate carboxylase; PFK, ATP-dependent phosphofructokinase; PFP, PPi-dependent phosphofructokinase; PGAM, phosphoglycerate mutase; PGK, phosphoglycerate kinase; PGM, phosphoglucomutase; PK, pyruvate kinase; PLS-DA, partial lest squares discriminant analysis; RbcL, rubisco large subunit; RbcS, Rubisco small subunit; RPI, ribose 5-phosphate isomerase; RT-qPCR, reverse transcription quantitative real time polymerase chain reaction; SCL, succinyl-CoA ligase; SuSy1, sucrose synthase 1; SuSy3, sucrose synthase 3; TCA, tricarboxylic acid cycle; UDP, uridine diphosphate; UTP, uridine triphosphate; VIP, variable importance in the projection

## Additional files

Additional file 1: Table S1. Primers used in real-time PCR analysis. (XLSX 11 kb) Additional file 2: Figure S1. Representative 2-DE maps of *E. grandis* bark proteins. (A) Bark summer proteins map. (B) Bark winter proteins map. Arrows indicate differentially expressed spots. Three biological replicates were used for each season. (TIF 912 kb) Additional file 3: Table S2. All metabolites identified in *E. grandis* bark. (XLSX 14 kb)
