# Supplementary figures and images for: Integrated analysis of gene expression from carbon metabolism, proteome and metabolome, reveals altered primary metabolism in Eucalyptus grandis bark, in response to seasonal variation
Source: BMC Plant Biol. 2016 Jul 1;16:149. doi: 10.1186/s12870-016-0839-8 (PMC4929727; doi:10.1186/s12870-016-0839-8)

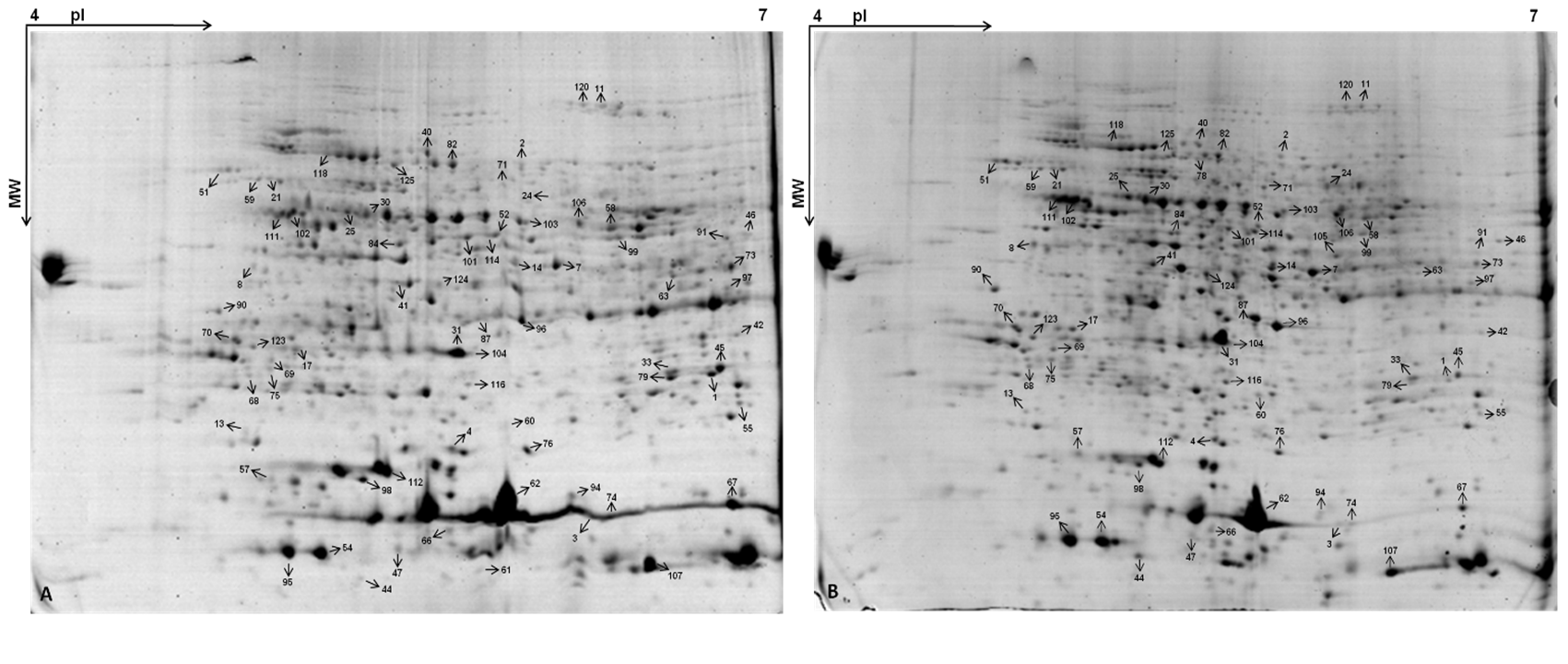

Supplement: Additional file 2: Figure S1. — Representative 2-DE maps of E. grandis bark proteins. (A) Bark summer proteins map. (B) Bark winter proteins map. Arrows indicate differentially expressed spots. Three biological replicates were used for each season. (TIF 912 kb) [file 12870_2016_839_MOESM2_ESM.tif]
